# Editorial: Abiotic Stress Signaling in Plants: Functional Genomic Intervention

**DOI:** 10.3389/fpls.2016.00681

**Published:** 2016-05-20

**Authors:** Girdhar K. Pandey, Amita Pandey, Manoj Prasad, Maik Böhmer

**Affiliations:** ^1^Department of Plant Molecular Biology, University of Delhi South CampusNew Delhi, India; ^2^National Institute of Plant Genome ResearchNew Delhi, India; ^3^Institute for Biology and Biotechnology of Plants, Westfälische Wilhelms UniversitätMünster, Germany

**Keywords:** signal transduction, functional genomics, genomics, crop production, abiotic stress

The major challenge before the plant scientists is to develop strategies to increase the productivity of crop plants so as to feed the rapidly increasing global population. The increased anthropogenic activities have resulted in radical decrease of fertile land available for agricultural practices, and in addition, the impact of climate change, which poses environmental stress resulting in drastic decrease in crop productivity are major concerns for plant biologists.

The sessile nature of plants has naturally evolved sophisticated molecular mechanisms to sense and respond to stress conditions, which can result in stress tolerance or stress avoidance. Signal transduction networks form a larger proportion of this complex machinery, which enable the plants to sense the stress signals followed by transduction and finally generation of the response. A large number of signaling molecules have been identified and shown to be involved in perception and signal transduction pathways. Similar to animals, many of the signal transduction pathways are conserved in plants forming a plethora of proteins such as receptors, G-proteins, kinases, phosphatases, transcription factors, channels and transporters (Hirt and Shonozaki, 2003; Pareek et al., 2010; Pandey, 2012, 2013). Despite the high conservation in signaling pathways, plants have also evolved certain novel signaling mechanisms where several unique components have been identified that are not found in their animal counterpart.

The complex interplay of single and multiple signal transduction networks allows plants to respond to these stimuli. In addition, these networks are responsible for normal growth and development, and communication with the environment under particular growth condition. At the same time, these complex-signaling circuitries also enable plants to develop memory, “intelligence” and behavior despite the lack of a definite nervous system (Baluška et al., 2006; Pandey, 2013, 2015).

In the last decade, genetic and biochemical approaches have facilitated the identification of numerous signaling pathway components in Arabidopsis. In addition, generation of a linear as well as compound signaling response has been shown in response to a particular stress stimulus. The compound response leads to crosstalk within major plant signaling pathways and is often associated with the plant's unique ability to combat multiple environmental stress conditions (Yoshika and Shinozazaki, 2009; Pareek et al., 2010; Pandey, 2015).

Identification and characterization of functional units involved in signaling cascades has further enabled plant biologists to understand the flow of information in response to a particular stimulus. These efforts are further enabled with the availability of whole genome sequences of many model as well as crop plants. Besides, a dramatic progress and use of high-throughput approaches have instigated a deeper understanding of cellular response machinery.

In the post-genomic era, a significant part of new experimental knowledge is provided by the progress in “Omics” based approaches such as transcriptomics, proteomics, metabolomics, interactomics and phenomics in several model organisms. These approaches have paved the foundation for “Functional Genomics”, which aims at the identification of genes and delineation of their functions. For the first time, this has allowed the estimation and simulation of molecular network inside the cells. In addition, this affords a cell-wide view of the regulatory and metabolic processes involved in translation from the protein to metabolite level to mount a response or phenotype against specific stimulus (Pandey, 2015).

In an attempt to understand stress mediated signal transduction in plants, we have conceived this special issue on “*Abiotic stress signaling in plants: Functional Genomics intervention”* to collate and present the current state of knowledge in this field. With an overwhelming response from several leaders in the field of stress signaling, a total of 49 articles (opinion, hypothesis, mini review, review and original article) have been published in this special issue. All these articles are categorized in six different sections. The first section, “Genomics and functional genomics approaches” is comprised of five articles. This section mainly describes the application of different functional genomics approaches to understand the stress signaling pathways and components. The review article by Das et al. describes the implication of genomics and functional genomics methodologies in understanding the salt tolerance response in rice, with a greater emphasis on integration of “Omics” based approaches to generate future crops, which can tolerate high salt level in soil without compromising yield and productivity. Following this, Jain has elaborated the use of the genome-editing tool, CRISPR-Cas9, to generate abiotic stress tolerant crops as an opinion article. Budak et al. presents a review article on the role of genetic to functional genomics approaches in improving the drought tolerance trait in wheat. Usage of Omics-based approaches in understanding plant physiology under the purely anthropogenic stress conditions, space and microgravity, is elaborated in the review by Schüler et al. The improvement of crops by systematic data integration of whole life cycle of crop plants in the field condition is presented in the mini-review by Mochida et al. They discuss how the integration of different datasets generated for population genomics, chronological omic analyses, and computer-aided molecular network prediction, can result in a holistic understanding of crop phenology that can be studied in the field to prevent yield shortfall as a result of environmental fluctuations due to climate change.

The second section covers “Plant hormone and abiotic stress responses” and encompasses six articles that focus on the different aspects of regulation of stress-mediated responses by phytohormones. Phytohormones are crucial chemical messengers acting locally or systemically to regulate plant growth, development, and adaptation to environmental conditions. Three articles in this section elaborate the functional role of jasmonic acid (JA) and its signaling components in regulation of abiotic stress responses. The review article by Sharma and Laxmi describes the biosynthesis and signal transduction pathway and its role in regulation of temperature stress. Another review by Riemann et al. discusses the role of jasmonates hormonal network in salinity and drought stress responses. The research article by Singh et al. focuses on the elucidation of the role of JAZ repressor proteins under different mineral nutrient deficiencies in rice and chickpea using expression analysis. Simontacchi et al present a review article on the role of nitric oxide (NO) in regulating the abiotic stresses like low mineral nutrient supply, drought, salinity and high UV-B radiation responses. ABA is an extensively studied phytohormone, which regulate both abiotic as well as biotic stress responses in plants.

The research article by Kim et al. describes the development of transient gene expression for rice protoplasts (TGERP), which provides a powerful tool for functional genomic analysis of rice and other crop plants. In addition, the TGERP approach could unveil the complexities in different genes and gene networks involved in regulating stress signaling in plants. The genome-wide survey and expression analysis of AUX/IAA genes in chickpea and soybean by Singh and Jain accounts for the possible role of these genes in auxin signaling during development and abiotic stress conditions. The last article of this section by Singh et al. discusses the role of salicylic acid (SA) in reducing the toxicity caused by arsenic, mainly by inhibiting the translocation of arsenic from root to shoot in rice plants.

The third section, “Signal transduction components and abiotic stress responses,” is comprised of 9 articles that emphasize different aspects of the signaling machinery and their components involved in abiotic stresses. Reactive oxygen species (ROS) act as crucial signaling molecules in programmed cell death and defense against pathogens in eukaryotic cells. Both biotic and abiotic stress signaling and responses are mediated by ROS; however, specificity is maintained in these two different major stresses. The review article by Jalmi and Sinha elaborates the differences and similarities of ROS mediated mitogen activated protein kinase (MAPK) signaling in response to abiotic and biotic stresses. The next three articles (two reviews and one research article) elaborate the role of calcium (Ca^2+^) in abiotic stress signaling. Ca^2+^ is considered as the ubiquitous and magic bullet in cell signaling and regulating diverse biological responses in both plant and animal systems. The review articles by Zeng et al. and Virdi et al. comprehensively explain the role of the important Ca^2+^ sensors, calmodulin, and calmodulin-like proteins, in regulating diverse stress responses, especially abiotic factors in plants. Beside calmodulin, calcineurin B-like protein (CBL) is also one of the major Ca^2+^ sensors involve in regulating diverse stress physiology in plants. CBLs interact with a Ser/Thr protein kinase family called CBL-interacting protein kinases (CIPKs) and this CBL-CIPK module regulate diverse physiological processes, especially abiotic stress responses (Pandey et al., 2014; Sanyal et al., 2015).

The article by Meena et al. highlights the role of CIPK25 of chickpea in regulating the root growth and responses to salt and dehydration stress. The role of G-protein components also has been extensively studied in both animal and plant signaling processes including stress physiology. Despite high similarities in G-protein component mediated signaling processes in animal and plants, they also show considerable variances in the signaling mechanism. Two articles by the same research group (Chakraborty et al. and Jangam et al.) emphasize the role of G-protein signaling components; GCR1 and GPA1 (Arabidopsis) and RGA1 (Rice D1) during multiple abiotic stresses by global transcriptomic approaches. Chandna et al. present the transcriptional differentiation of 14-3-3 proteins in *Brassica rapa* in response to stress and development. These 14-3-3 proteins are highly conserved protein present in all eukaryotes and involved in protein-protein interaction and hence regulate signal transduction processes. In an interesting article by Kaur et al. the role of methyl glyoxal (MG), a toxic compound produced as by-product of glycolysis is speculated to be a stress signal based on global gene expression profiling experiments in rice. The gene expression regulation of two-component system (TCS), which comprised of Histidine kinases (HKs), Histidine phosphotransfer protein (HPTs), and Response regulators (RRs) in abiotic stresses, tissue-specific manner and diurnal rhythm is presented by Singh et al. in both Arabidopsis and rice. The article by Jamsheer and Laxmi bring forward the expression analysis of FCS-like Zinc Finger (FLZ) genes in abiotic stress, sugars, and cellular energy level. FLZ proteins were found to interact with sucrose non-fermenting (SNF) related kinases (SnRK) and speculated to be involved in regulating the cellular energy and stress responses in plants.

In the fourth section, seven articles highlighting the role of transcription factors (TFs) in abiotic stress responses are compiled. The first article by Shao et al. addresses the role of NAC (NAC acronym is derived from three earliest characterized proteins with a particular NAC domain from petunia NAM, no apical meristem from *Arabidopsis*, ATAF1/2 and CUC2 cup-shaped cotyledon). These TFs are involved in regulating the plant development and stress responses including biotic and abiotic stresses.

Another review article by Singh and Laxmi describes the role of various TFs in regulating the drought responses in plants. Beside TFs, there are other factors, which either increase or decrease the rate of transcription, and the mediator complex is one such example. The review article by Samanta and Thakur describes in detail the function of the mediator complex in integration of diverse signaling pathway including stress responses in plants. Nguyen et al. has undertaken a differential gene expression analysis in drought sensitive and tolerant cultivars of chickpea and identified a subset of potential chickpea NAC transcription factors, which could be used for generation of drought tolerant chickpea with improved productivity under drought stress. The next two research articles by Yan et al. and Muthamilarasan et al. focus on the plant specific WRKY transcription factors from cotton, foxtail millet (*Setaria italica*) and green foxtail (*S. viridis*) in regulating stress responses in plants. Muthamilarasan et al. used global transcriptomic analysis to identify the potential WRKY transcription factors involved in abiotic stresses, and have pinpointed potential candidates for further functional characterization. Yan et al. identified the functional role of one of the WRKY members of cotton, which negatively regulates drought tolerance and positively regulates the resistance to *R. solani*.

The fifth section deals with “Gene expression analysis and response under stress conditions” and is comprised of 10 articles. After extensive research in the field of small non-coding RNA, it is well appreciated that these are key modulators of post-transcriptional gene silencing and are involved in regulating a diverse array of biological responses. The first four articles (Sablok et al.; Das et al.; Tripathi et al.; Ebrahimi et al.) present various aspects of small non-coding RNAs such as miRNA, siRNA and ta-siRNA in regulating biological processes ranging from germination and development to abiotic stress responses in plants. The detail transcriptomic profiling has been undertaken in Indian mustard (*Brassica juncea*), which is one of the major oil crops for the Indian population (Sinha et al.; Srivastava et al.).

The study by Sinha et al. present the *de novo* global gene expression analysis of cold-stressed Indian mustard siliques during pod filling to delineate the signaling network. The study by Srivastava et al. elaborates the identification of key candidate genes and regulatory network of arsenic-stressed rice seedlings by whole genome transcriptomic approach. A comparative root transcriptomic study in drought-response contrasting cultivars of soybean by Ha et al. identified differentially expressed genes encoding osmoprotectant biosynthesis-, detoxification-, or cell wall-related proteins, kinases, transcription factors and phosphatase 2C proteins. The genome-wide transcriptional and metabolic effects of low level xenobiotic stress on an important grass species, *Lolium perenne*, was studied by Serra et al. which revealed the involvement of multiple signaling cascades such as metabolic and phytohormone regulated pathways in xenobiotic stress. The comprehensive identification and expression analysis of membrane bound tetraspannin protein in rice was undertaken by Mani et al. Their detailed expression analysis hints to the possible involvement of tetraspanin proteins in cellular signaling and other biological processes in plant during different developmental stages and under different abiotic stresses. The last article of this section by Shukla et al. describes the role of natural variation in expression of regulatory and detoxification related genes under low phosphate and arsenic stress in Arabidopsis.

The sixth and last section entitled “Diverse aspect of abiotic stress responses” is comprised of 11 articles. These articles were organized with the aim of educating the readers on different proteins or processes related to diverse abiotic stresses. The first article by Khatri and Mudgil hypothesizes the functional role of N-MYC DOWN REGULATED-LIKE (NDL) proteins in abiotic stress responses involves regulation of microtubule organization. The review article by Jagadish et al. provides arguments for designing a target phenotype to mitigate abiotic stresses during pre- and post-anthesis in cereals with a focus on hormonal balances regulating stay-green phenotype versus remobilization. Another review article by Driedonks et al. elaborately describes the multi-level interaction of heat shock factors, heat shock proteins and redox system to impart acclimation or heat tolerance in crop plants.

In natural conditions, plants are constantly exposed to multiple abiotic stresses or a combination of biotic and abiotic stresses. This aspect is well elaborated by Pandey et al. where they discuss the details of molecular and physiological mechanisms of plant's responses to multiple individual stresses and stress combinations that could lead to develop strategies to raise crop varieties with broad-spectrum stress tolerance. Cyclophillins are the proteins reported to bind to the immunosuppressant drug cyclosporin A in animal. Cyclophillin proteins are also known to exist in plants as multigene families and many of these also bind to the immunosupressant drug. Lee et al. have functionally characterized one of these family members, OsCYP21-4 from rice, which is localized exclusively in Golgi bodies. OsCYP21-4 overexpression confers salt and oxidative stress tolerance in rice. Small heat shock proteins (sHSP) are a diverse group of proteins that are mostly expressing in the seeds of plants. Kaur et al. identified one of the sHSP proteins from rice, OsHSP18.2, which is differentially expressed in seed and aging tissues. OsHSP18.2 is involved in seed vigor, longevity, and improve seed germination under abiotic stress conditions. Zhang et al. identified an aquaporin gene from cotton plant termed as tonoplast intrinsic protein (TIP), GmTIP2;3. Subcellular localization shows that GmTIP2;3 is plasma membrane localized protein and its heterologous expression improves osmotic stress tolerance in yeast. The expression of proline accumulation and metabolism-related genes under salt stress was analyzed by Wang et al. in *Kosteletzkya virginica*. In their expression analysis, the role of *KvP5CS1*, the key gene involved in proline accumulation, was found to be significant in leaves under salt stress. The study by Sinha et al. revealed the identification of proteins containing the universal stress protein (USP) domain in pigeonpea in drought stress. The evolutionary conservation of arginine-rich tandem zinc-finger protein (RR-TZF), AtTZF1, 2 and 3 homolog regulation under salt was found in distant species such as Arabidopsis, durum wheat and lower plants such as *Chlamydomonas* and moss by Orso et al. The last article of this section by Sinha et al. validated the selection of most suitable housekeeping reference genes as endogenous controls in gene expression studies in pigeonpea under salt and heat stress conditions.

Overall, most of the articles in this special issue emphasized the genomics and functional genomics aspects of various signal transduction components, pathways, and gene regulatory networks and their potential in developing climate resilient crop plants by multiple approaches to tame the major challenge of crop loss due to abiotic stresses in the field. Indeed, not all the aspects of signal transduction components and pathways regulating the abiotic stress signaling and responses could be covered but an in-depth insight can be extrapolated for many of these signaling pathways. A detailed discussion of several different aspects of abiotic stress signaling also put forward a large number of unanswered questions such as how plants respond to multiple stress factors in the field and how specificity and overlap is maintained in the response? One of the foremost challenges to plant biologists is how plants can be engineered to tackle the problem of crop loss due to both biotic and abiotic stresses in the field condition. Indeed, the advanced multidisciplinary approaches of genomics and functional genomics, biochemistry and physiology hold the key to address these important questions in the near future.

## Author contributions

GP wrote the article; GP, AP, MP, and MB have revised the manuscript.

### Conflict of interest statement

The authors declare that the research was conducted in the absence of any commercial or financial relationships that could be construed as a potential conflict of interest.
